# Performance of Two Screening Questionnaires for Inflammatory Arthritis in Patients with Inflammatory Bowel Disease

**DOI:** 10.1155/2018/8618703

**Published:** 2018-05-08

**Authors:** Rubén Queiro, Sergio Rodríguez-Caminero, Sabino Riestra, Ruth de Francisco, Isabel Pérez-Martínez, Javier Ballina

**Affiliations:** ^1^Rheumatology Division, Hospital Universitario Central de Asturias (HUCA), Oviedo, Spain; ^2^Gastroenterology Division, IBD Unit, Hospital Universitario Central de Asturias (HUCA), Oviedo, Spain

## Abstract

Musculoskeletal symptoms are the most common extraintestinal manifestations of inflammatory bowel disease (IBD). An essential step in the management of these patients is to establish referral algorithms through the use of appropriate screening tools. Our objective was to evaluate the performance of two simple questionnaires to detect inflammatory arthritis (IA) in patients with IBD. Two questionnaires, one for detecting axial IA and the other for peripheral IA, were tested among 112 IBD unselected consecutive patients of both sexes, aged 18–45 years. The study period was from January to December 2016. Each questionnaire was composed of three simple questions. If the patient answered affirmatively at least to two of the three questions, the questionnaire was considered positive. Clinical diagnosis of IA based on an expert's opinion was the reference gold standard. To obtain a weighted value of sensitivity and specificity, likelihood ratio (LR) values were calculated. Twenty-seven percent of the patients were considered positive responders to the axial questionnaire while 32% were considered positive responders to the peripheral questionnaire. Twenty-four patients (21.4%) were diagnosed with axial IA, whereas 26% had peripheral IA. The axial questionnaire yielded a sensitivity of 87.5% (67.6–97.3), specificity of 89.8% (81.5–92.2) and LR+ of 8.6 (4.5–16.2). For the peripheral questionnaire, these values were 82.8% (64.2–94.2), 87.4% (79–93.3), and 6.6 (3.8–11.4), respectively. Both questionnaires showed an adequate screening capacity for IA in patients with IBD. Their specificity, together with their simplicity, can make them suitable detection tools in gastroenterology and general medicine consultations.

## 1. Introduction

Inflammatory bowel disease (IBD) is a chronic immune-mediated disease that affects the gastrointestinal tract. It is comprised of two subtypes: Crohn's disease (CD) and ulcerative colitis (UC). Both are thought to result from complex stochastic interactions between aberrant immune responses to gut luminal microbes in genetically susceptible individuals who are exposed to environmental risk factors [1–3]. IBD is most common in North America and western and northern Europe, where incidence rates of UC and CD range from 2.2 to 24.3 per 10^5^ person-years [3].

Musculoskeletal symptoms (MSs) are the most common extraintestinal manifestations in patients with IBD. Many of these IBD-associated MSs belong to the broader concept of spondyloarthritis (SpA) [4–7]. The prevalence of inflammatory rheumatic manifestations is highly variable in IBD. In a recent meta-analysis, the pooled prevalence rates of rheumatic diagnoses were as follows: sacroiliitis, 10%; ankylosing spondylitis, 3%; peripheral arthritis, 13%; enthesitis, 1% to 54%; and dactylitis, 0% to 6% [8]. However, when all these manifestations of the SpA spectrum are grouped together, more than one-third of IBD patients show the SpA features included in the new Assessment of SpondyloArthritis International Society (ASAS) criteria [9, 10].

Some IBD patients may show peripheral/axial arthritis that sometimes runs a parallel course with gut inflammation, while in others, joint and gut inflammation run independent courses [4–7, 11]. In daily practice, symptoms of SpA, either axial or peripheral, are not adequately recognized by either patients or gastroenterologists [9]. To most patients, the relationship between joint and gut symptoms is unknown, and gastroenterologists do not always specifically ask patients about joint involvement. Consequently, patients with symptoms of SpA may be underdiagnosed and effective treatment may be delayed. In a study that included 350 unselected patients with IBD, half of the patients who reported at least one musculoskeletal SpA feature never visited a rheumatologist. Axial or peripheral SpA was diagnosed in 58% of the patients who were examined by a rheumatologist, and in 21%, another rheumatic disorder was diagnosed [9].

Therefore, the collaboration between rheumatologists and gastroenterologists should be strengthened, as both gut and joint inflammations can seriously deteriorate the quality of life of patients. In many cases, the therapeutic decision process is common to both conditions [12, 13]. A key part of this collaboration is to establish appropriate derivation algorithms from one specialty to another, without these algorithms implying an excessive assistance load for any of the specialties involved [12, 13]. Ideally, these tools should be valid and easy to use in routine clinical practice.

We aimed to evaluate the diagnostic performance of two screening questionnaires for inflammatory arthritis (IA) in subjects with IBD. The simplicity of these questionnaires could lead to improved care for patients with IBD and suspected SpA.

## 2. Patients and Methods

The participants were prospectively recruited from a single university institution in northern Spain. The inclusion criteria were patients of both sexes aged 18–45 years, a verified diagnosis of IBD (based on endoscopic, laboratory, and histological findings), and ability to provide written informed consent. The exclusion criteria were patients with a known rheumatologic or musculoskeletal condition, patients involved in a labor litigation, patients awaiting recognition of incapacity, or those with temporary or permanent disabilities due to their IBD, and those found to be unable to comply with the study procedures. The inclusion period was from January 2016 to December 2016.

All the patients provided their informed written consent. In accordance with the Spanish recommendations, the study was approved by the Clinical Research Ethics Committee of Hospital Universitario Central de Asturias (reference number HUCA 67/14) and was conducted in accordance with the principles of the Declaration of Helsinki for studies on humans.

This study was developed in several steps. First, two rheumatologists with expertise in SpA agreed on three questions for the detection of axial arthritis and three questions for the detection of peripheral arthritis.

The three questions agreed upon for peripheral arthritis were as follows:

- Do you have joint pain?
- When waking up in the morning, do you notice stiffness in your joints for a time equal to or greater than 30 minutes?
- Do you have or have you had any swollen joint?

The overall questionnaire response was considered positive if the patient answered affirmatively to at least two of the three questions.

The three questions agreed upn for axial arthritis were as follows:

- Do you have back pain?
- When waking up in the morning, do you notice stiffness in your back for a time equal to or greater than 30 minutes?
- Do you have or have you had back pain that wakes you up or interrupts your sleep?

The overall questionnaire response was considered positive if the patient answered affirmatively to at least two of the three questions.

To avoid including patients with degenerative joint diseases that could bias the positivity of the questionnaire responses, only adult patients aged ≤45 years were included. This age limit is the same as that used in the ASAS criteria for SpA [10].

The clinical part of the study consisted of detailed clinical history, family medical history, physical examination, and laboratory and imaging tests (except ultrasonography (US) and according to the criteria of the evaluating physician). The final diagnosis of IA, either axial or peripheral, was established by a rheumatologist with extensive experience in SpA.

Second, all the patients underwent a US study of peripheral joints and entheses. This part of the study was conducted by an expert in musculoskeletal US, who was blinded to the clinical part of the study. The US examination was performed with MyLab 70XVG (Esaote S.p.A., Genova, Italy). Details of this US protocol have been published elsewhere [14, 15]. Enthesitis, synovitis, synovial effusion, synovial hypertrophy, positive power Doppler (PD) signal, and so forth were defined in accordance with the standards provided by OMERACT's musculoskeletal US working group [16].

### 2.1. Statistical Methods

A descriptive statistical analysis of all the variables was performed, including central tendency and dispersion measures for continuous variables and absolute and relative frequencies for categorical variables. Student's *t*-test, Mann–Whitney *U* test, or Kruskal-Wallis *H* test was used to compare quantitative variables, and Pearson's chi-square or Fisher's exact test was used for qualitative variables. Tests were two-tailed with a significance level of 5%. Data were analyzed using the SPSS v19.0 statistical software.

The sample size was calculated to achieve an accuracy of 10% using a normal bilateral asymptotic 95% confidence interval (CI), assuming a sensitivity of 80%. To evaluate sensitivity and specificity, both questionnaires were tested in the whole study population and among 110 healthy subjects. The healthy control population was matched by age (34 ± 6.8 yrs) and sex (44.5% females and 55.5% males) with the study population. Subjects with a personal history of inflammatory rheumatic diseases or a family history of psoriasis or inflammatory rheumatic processes were excluded as controls.

Test-retest reliability, sensitivity, specificity, positive/negative LR, and positive/negative predictive values were calculated for both questionnaires. Intrarater reliability (Cohen's kappa) was calculated for the US studies conducted in the study population and in 30 healthy age- and sex-matched controls.

## 3. Results

Of the 112 patients, 48 (42.9%) were women and 64 (57.1%) were men, with a mean age of 33 ± 7.2 years. Forty-four cases were UC, and 68 were CD. The mean duration of IBD was 8 ± 5.6 years. As for treatments, 42 patients received biological therapy, and 51 received classical immunosuppressants (mostly thiopurines). We found no significant differences with respect to age, sex, duration of illness, level of education, BMI, or IBD family history between CD and UC.  Table 1 represents the main disease characteristics of the study population.

**Figure 1:**
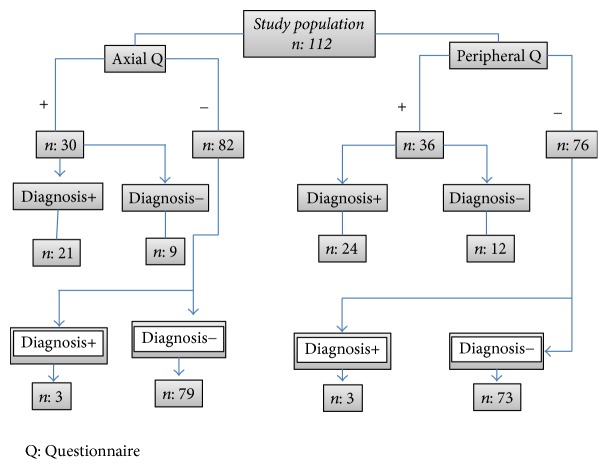
Flow chart according to the answers to the questionnaires.

Based on the questionnaire responses, 27% of the patients were considered positive responders to the axial questionnaire and 32% were considered positive responders to the peripheral questionnaire. Twenty-four patients (21.4%) were diagnosed with axial IA, whereas 26% had peripheral IA. Sensitivity, specificity, and LR+ values for the axial questionnaire were 87.5%, 89.8%, and 8.6, respectively, whereas for the peripheral questionnaire, these values were 82.8%, 87.4%, and 6.6, respectively. In the control population, specificity for the axial and peripheral questionnaires was 90.7% (83.6–95.5) and 89.6% (82.4–94.7), respectively. The test-retest reliability revealed an excellent CCI of 0.94 (0.92–0.99) for both questionnaires.  Figure 1 represents the flow chart of the study population according to questionnaire responses.

These results were not affected by exposure to systemic treatments. The LR+ of the axial arthritis questionnaire was 7.6 (3.8–16.7) in the patients not exposed to systemic therapies and 8.4 (4.4–15.6) in those exposed to these drugs. For the peripheral arthritis questionnaire, the LR+ value was 6.7 (3.8–13.8) in patients not exposed to systemic therapies and 7.4 (4.3–12.8) in those exposed to systemic therapies.

Intrarater reliability was excellent in both US substudies (0.93 [0.92–0.98]). More US changes were found in the study population with respect to the control group. The percentage of patients with synovial effusion and/or synovial hypertrophy was greater in the study population than in the control group (31% versus 10%, *p* = 0.001). More structural changes were detected in the patients' entheses than in those of the controls (41.9% versus 13.3%, *p* = 0.0001). More patients (40.2%) had findings of enthesitis and/or active synovitis (PD positive signal) than the controls (3.3%; *p* < 0.0001). The sensitivity, specificity, and LR+ of US examination for clinical diagnosis of IA were 50.5%, 64.9%, and 1.6, respectively (*p* = 0.046).

We found no statistically significant associations between the positive questionnaire responses and the positive US findings. However, when the responses to both questionnaires were negative, the US findings were also negative, with a specificity of 89.2% (79.1–95.6).  Table 2 summarizes the reliability of the questionnaire responses and US findings.

## 4. Discussion

In this study, we verified that two simple questionnaires were consistent with regard to the detection of IA in patients with IBD, with high sensitivity and specificity. On the other hand, in this unselected population, the prevalence of IA with features of SpA was high. Almost one-fourth of the study population had IA according to the criteria of a rheumatologist expert in SpA. These data are in line with prevalence data from earlier studies, thus reinforcing the reliability of both questionnaires [4–7].

The two questionnaires tested here showed a high sensitivity and specificity, so their predictive capacity for IA may be useful in daily practice for gastroenterologists and family medicine doctors, thus leading to improved referrals for rheumatology consultations. Such ability depends largely on the pretest probability of finding a true case of IBD-associated arthritis. Following the principles of the Bayes theorem, if the expected prevalence of IA in this population is intermediate-high (30–50%) according to the international literature, for a positive LR of approximately 6–9, which were the values obtained from our study, we would obtain posttest probabilities of arthritis of 70–80%. In other words, of 10 patients with IBD who might answer positively to either questionnaire, between 7 and 8 could actually have IBD-associated arthritis. In addition, if we take into account the fact that both questionnaires are simple and easy to implement in everyday practice, it is worthwhile to test their usefulness in other centers to obtain an external validation of these results. Together, both questionnaires yielded a high specificity (≈90%), which indicates that they have the potential to determine which patients should (or not) be sent to rheumatology appointments.

We found a high prevalence of US findings both in the joints and in the entheses of these patients. These findings were significantly higher than those found in the healthy control population matched by age and sex. On the other hand, the association between our US findings and the clinical questionnaire responses was weak or nonexistent. In other studies of musculoskeletal US performed in subjects with IBD, a high prevalence of subclinical US abnormalities has been found [17, 18]. Something similar has been published in patients with psoriasis without arthritis [19]. Therefore, our US data are in line with those of other studies in IBD or psoriasis and reflect the subclinical nature of most of them. The clinical significance of these findings will need to be elucidated in prospective studies with enough long follow-up. Moreover, the prevalence of these findings was not affected by exposure to systemic therapies (data not shown). When the responses to both questionnaires were negative, no positive findings were found on US (specificity close to 90%). This reinforces, once again, the high reliability of both questionnaires.

Questionnaires showed adequate sensitivity and specificity both in exposed patients and in those not exposed to systemic treatments. This is an unexpected finding. We could not make an exact correction according to the past or current exposure to systemic therapies. When stratifying patients according to their exposure to this type of treatment, comparisons were reduced to relatively small groups of patients, and this could have been associated with a decrease in the statistical power of the study (type II error). Therefore, this finding should be taken with caution.

Some weaknesses of the study should be highlighted. One is that the study was conducted in a single university center that generally addresses the most serious cases of IBD. Therefore, we do not know if these questionnaires would work equally well in other clinical settings. The clinical diagnosis of IA was based on the judgment of a physician expert in SpA, but it was not contrasted to other specialists' diagnoses. It also was not clearly tested against the ASAS criteria for axial or peripheral SpA [10]. Therefore, we did not generate information about its consistency. In any case, the ASAS criteria are for classification purposes and cannot replace the diagnosis made by an SpA expert [10, 20]. Having chosen a young population (age limit of 45 years), we do not know how these instruments would behave in populations of patients with IBD who are above 45 years of age. Finally, the questionnaires were taken as a whole, without item-by-item weighting.

Within the strengths of this study, the simplicity and comfort of the questionnaires for physicians and patients are worth mentioning. In fact, the test-retest reliability (performed with an average interval of 2 weeks) was very high for both. The fact that almost one-fourth of the patients in this series had IA (a figure very similar to that reported in other studies) can be assumed as adequate face and content validities for both questionnaires.

In summary, we tested the usefulness of two simple questionnaires for detecting arthritis in patients with IBD. Both were better than US for the aforementioned purpose. Validation of the results of this study in other clinical settings would be interesting.

## Figures and Tables

**Table 1:** Disease characteristics of the study population.

Disease feature	Study population *N*: 112
Age (yrs, mean ± SD)	33.12 ± 7.19
Disease duration (yrs, mean ± SD)	8 ± 5.6
BMI (Kg/m^2^)	23.3 ± 4.5
Women	48 (42.9%)
Men	64 (57.1%)
UC	44 (39.3%)
CD	68 (60.7%)
IBD family history	21 (18.7%)
Biologics	42 (37.5%)
Classic immunosuppressants	51 (45.5%)
Corticoids	11 (9.8%)
IBD surgery	28 (25%)
Diabetes	0 (0%)
Hypertension	0 (0%)
Dyslipidemia	2 (1.8%)
Smokers	24 (21.4%)
Alcohol	25 (22.3%)
Psoriasis	11 (9.8%)
Psoriasis family history	14 (12.5%)
Asthma	14 (12.5%)
Clinical enthesitis^*∗*^	5 (4.5%)
Dactylitis^*∗*^	3 (2.7%)

BMI: body mass index; UC: ulcerative colitis; CD: Crohn's disease; IBD: inflammatory boweldisease. ^*∗*^After clinical examination.

**Table 2:** Reliability parameters of the screening questionnaires.

Parameter	Axial Q versus clinical D	Per. Q versus clinical D	US versus clinical D
Sensitivity	87.5% (67.6–97.3)	82.8% (64.2–94.2)	50.5% (36.4–71.9)
Specificity	89.8% (81.5–92.2)	87.4% (79–93.3)	64.9 (53.2–75.4)
Positive LR	8.6 (4.5–16.2)	6.6 (3.8–11.4)	1.6 (1.1–2.4)
Negative LR	0.14 (0.05–0.4)	0.2 (0.09–0.4)	0.7 (0.5–1)
PPV	70% (50.6–85.3)	66.7% (49–81.4)	40% (25.7–55.7)
NPV	96.3% (89.7–99.2)	94.3% (87.2–98.1)	76.9% (64.8–88.5)

LR: likelihood ratio; Q: questionnaire; Per: peripheral; D: diagnosis; US: ultrasound; PPV: positive predictive value; NPV: negative predictive value.

## Data Availability

All the clinical data of this study are conserved in databases of the gastroenterology and rheumatology services of HUCA, Avda. de Roma s/n, 33011 Oviedo, Spain.
